# Supplementary material for: Modeling glioblastoma heterogeneity as a dynamic network of cell states
Source: Mol Syst Biol. 2021 Sep 16;17(9):e10105. doi: 10.15252/msb.202010105 (PMC8444284; doi:10.15252/msb.202010105)
Supplement: Supplementary file 6 — Source Data for Figure 5 [file MSB-17-e10105-s004.zip › Figure5A_sourcedata/GSEA_3071/hallmarks_stateV.GseaPreranked.1623742546329/HALLMARK_EPITHELIAL_MESENCHYMAL_TRANSITION.html]

Details for gene set HALLMARK\_EPITHELIAL\_MESENCHYMAL\_TRANSITION[GSEA]

|  || Dataset | state43071 |
| Phenotype | NoPhenotypeAvailable |
| Upregulated in class | na\_neg |
| GeneSet | HALLMARK\_EPITHELIAL\_MESENCHYMAL\_TRANSITION |
| Enrichment Score (ES) | -0.64091074 |
| Normalized Enrichment Score (NES) | -3.7834733 |
| Nominal p-value | 0.0 |
| FDR q-value | 0.0 |
| FWER p-Value | 0.0 |
Table: GSEA Results Summary

  

Fig 1: Enrichment plot: HALLMARK\_EPITHELIAL\_MESENCHYMAL\_TRANSITION      
 Profile of the Running ES Score & Positions of GeneSet Members on the Rank Ordered List

  

| PROBE | GENE SYMBOL | GENE\_TITLE | RANK IN GENE LIST | RANK METRIC SCORE | RUNNING ES | CORE ENRICHMENT || 1 | IGFBP2 |  |  | 8 | 1.922 | 0.0117 | No |
| 2 | IGFBP3 |  |  | 78 | 1.437 | 0.0083 | No |
| 3 | MCM7 |  |  | 88 | 1.400 | 0.0162 | No |
| 4 | FBLN1 |  |  | 95 | 1.383 | 0.0246 | No |
| 5 | CADM1 |  |  | 152 | 1.264 | 0.0224 | No |
| 6 | GAS1 |  |  | 154 | 1.263 | 0.0310 | No |
| 7 | TNC |  |  | 246 | 1.123 | 0.0211 | No |
| 8 | CDH2 |  |  | 255 | 1.115 | 0.0272 | No |
| 9 | ELN |  |  | 357 | 1.001 | 0.0146 | No |
| 10 | PFN2 |  |  | 680 | 0.759 | -0.0425 | No |
| 11 | SCG2 |  |  | 682 | 0.756 | -0.0375 | No |
| 12 | DPYSL3 |  |  | 691 | 0.750 | -0.0339 | No |
| 13 | PMEPA1 |  |  | 718 | 0.740 | -0.0338 | No |
| 14 | ENO2 |  |  | 728 | 0.731 | -0.0305 | No |
| 15 | APLP1 |  |  | 792 | 0.704 | -0.0378 | No |
| 16 | VEGFA |  |  | 926 | 0.649 | -0.0591 | No |
| 17 | COL1A2 |  |  | 1028 | 0.614 | -0.0744 | No |
| 18 | FBN2 |  |  | 1029 | 0.614 | -0.0702 | No |
| 19 | TGFBR3 |  |  | 1203 | 0.560 | -0.0998 | No |
| 20 | EFEMP2 |  |  | 1300 | 0.532 | -0.1147 | No |
| 21 | PDLIM4 |  |  | 1382 | 0.511 | -0.1269 | No |
| 22 | LRRC15 |  |  | 1458 | 0.491 | -0.1380 | No |
| 23 | SGCB |  |  | 1677 | 0.441 | -0.1772 | No |
| 24 | HTRA1 |  |  | 1958 | 0.395 | -0.2287 | No |
| 25 | FZD8 |  |  | 1961 | 0.394 | -0.2263 | No |
| 26 | MEST |  |  | 1980 | 0.391 | -0.2271 | No |
| 27 | CDH6 |  |  | 2101 | 0.374 | -0.2478 | No |
| 28 | ID2 |  |  | 2104 | 0.373 | -0.2456 | No |
| 29 | PCOLCE2 |  |  | 2215 | 0.361 | -0.2644 | No |
| 30 | ACTA2 |  |  | 2227 | 0.359 | -0.2641 | No |
| 31 | ABI3BP |  |  | 2402 | 0.334 | -0.2954 | No |
| 32 | SDC1 |  |  | 2491 | 0.323 | -0.3102 | No |
| 33 | ECM1 |  |  | 2564 | 0.315 | -0.3220 | No |
| 34 | SFRP1 |  |  | 2591 | 0.312 | -0.3249 | No |
| 35 | COLGALT1 |  |  | 2623 | 0.307 | -0.3288 | No |
| 36 | CXCL12 |  |  | 2633 | 0.306 | -0.3284 | No |
| 37 | PRRX1 |  |  | 2674 | 0.302 | -0.3341 | No |
| 38 | TIMP3 |  |  | 2826 | 0.286 | -0.3613 | No |
| 39 | CALU |  |  | 3001 | 0.269 | -0.3932 | No |
| 40 | MMP2 |  |  | 3234 | -0.257 | -0.4363 | No |
| 41 | GPX7 |  |  | 3263 | -0.261 | -0.4399 | No |
| 42 | LAMA2 |  |  | 3449 | -0.289 | -0.4737 | No |
| 43 | PPIB |  |  | 3507 | -0.301 | -0.4827 | No |
| 44 | ITGB3 |  |  | 3508 | -0.301 | -0.4806 | No |
| 45 | TPM4 |  |  | 3591 | -0.314 | -0.4943 | No |
| 46 | GJA1 |  |  | 3602 | -0.316 | -0.4941 | No |
| 47 | ITGB1 |  |  | 3614 | -0.317 | -0.4940 | No |
| 48 | TNFAIP3 |  |  | 3691 | -0.330 | -0.5065 | No |
| 49 | VIM |  |  | 3830 | -0.354 | -0.5307 | No |
| 50 | SERPINE2 |  |  | 3951 | -0.377 | -0.5514 | No |
| 51 | COL5A3 |  |  | 3975 | -0.383 | -0.5532 | No |
| 52 | CD59 |  |  | 4024 | -0.394 | -0.5598 | No |
| 53 | JUN |  |  | 4135 | -0.418 | -0.5782 | No |
| 54 | ITGAV |  |  | 4207 | -0.442 | -0.5889 | No |
| 55 | FSTL1 |  |  | 4262 | -0.460 | -0.5961 | No |
| 56 | COL8A2 |  |  | 4277 | -0.467 | -0.5956 | No |
| 57 | MFAP5 |  |  | 4343 | -0.485 | -0.6049 | No |
| 58 | IL15 |  |  | 4346 | -0.486 | -0.6019 | No |
| 59 | NOTCH2 |  |  | 4355 | -0.488 | -0.6001 | No |
| 60 | GADD45A |  |  | 4399 | -0.500 | -0.6050 | No |
| 61 | FERMT2 |  |  | 4445 | -0.519 | -0.6101 | No |
| 62 | VCAN |  |  | 4491 | -0.537 | -0.6151 | No |
| 63 | BMP1 |  |  | 4559 | -0.569 | -0.6241 | No |
| 64 | ITGA5 |  |  | 4580 | -0.576 | -0.6240 | No |
| 65 | LOXL1 |  |  | 4607 | -0.593 | -0.6250 | No |
| 66 | LOXL2 |  |  | 4624 | -0.605 | -0.6239 | No |
| 67 | FSTL3 |  |  | 4651 | -0.619 | -0.6247 | No |
| 68 | NTM |  |  | 4695 | -0.643 | -0.6286 | No |
| 69 | CAPG |  |  | 4709 | -0.650 | -0.6266 | No |
| 70 | LGALS1 |  |  | 4784 | -0.699 | -0.6361 | Yes |
| 71 | BDNF |  |  | 4795 | -0.707 | -0.6331 | Yes |
| 72 | RHOB |  |  | 4820 | -0.731 | -0.6327 | Yes |
| 73 | PLOD1 |  |  | 4854 | -0.757 | -0.6339 | Yes |
| 74 | THY1 |  |  | 4874 | -0.781 | -0.6322 | Yes |
| 75 | P3H1 |  |  | 4912 | -0.825 | -0.6337 | Yes |
| 76 | TGM2 |  |  | 4928 | -0.842 | -0.6308 | Yes |
| 77 | TGFB1 |  |  | 4944 | -0.861 | -0.6277 | Yes |
| 78 | FLNA |  |  | 4948 | -0.862 | -0.6224 | Yes |
| 79 | CALD1 |  |  | 4960 | -0.877 | -0.6184 | Yes |
| 80 | MATN3 |  |  | 4961 | -0.878 | -0.6124 | Yes |
| 81 | NNMT |  |  | 5004 | -0.942 | -0.6140 | Yes |
| 82 | IL32 |  |  | 5006 | -0.946 | -0.6077 | Yes |
| 83 | QSOX1 |  |  | 5018 | -0.968 | -0.6031 | Yes |
| 84 | COL7A1 |  |  | 5041 | -1.021 | -0.6003 | Yes |
| 85 | SERPINH1 |  |  | 5059 | -1.050 | -0.5964 | Yes |
| 86 | EMP3 |  |  | 5084 | -1.113 | -0.5934 | Yes |
| 87 | LOX |  |  | 5092 | -1.128 | -0.5869 | Yes |
| 88 | GLIPR1 |  |  | 5109 | -1.177 | -0.5819 | Yes |
| 89 | COL11A1 |  |  | 5110 | -1.178 | -0.5738 | Yes |
| 90 | COL5A2 |  |  | 5112 | -1.189 | -0.5658 | Yes |
| 91 | TIMP1 |  |  | 5134 | -1.251 | -0.5612 | Yes |
| 92 | TGFBI |  |  | 5136 | -1.259 | -0.5527 | Yes |
| 93 | MMP14 |  |  | 5146 | -1.297 | -0.5455 | Yes |
| 94 | GADD45B |  |  | 5155 | -1.335 | -0.5378 | Yes |
| 95 | SERPINE1 |  |  | 5161 | -1.377 | -0.5293 | Yes |
| 96 | VEGFC |  |  | 5177 | -1.443 | -0.5222 | Yes |
| 97 | PLAUR |  |  | 5185 | -1.479 | -0.5134 | Yes |
| 98 | COL12A1 |  |  | 5186 | -1.484 | -0.5032 | Yes |
| 99 | THBS1 |  |  | 5197 | -1.538 | -0.4945 | Yes |
| 100 | COL4A2 |  |  | 5198 | -1.545 | -0.4838 | Yes |
| 101 | MATN2 |  |  | 5200 | -1.557 | -0.4733 | Yes |
| 102 | ITGA2 |  |  | 5201 | -1.557 | -0.4625 | Yes |
| 103 | TPM1 |  |  | 5215 | -1.643 | -0.4537 | Yes |
| 104 | BGN |  |  | 5216 | -1.654 | -0.4423 | Yes |
| 105 | BASP1 |  |  | 5218 | -1.662 | -0.4310 | Yes |
| 106 | IGFBP4 |  |  | 5226 | -1.725 | -0.4205 | Yes |
| 107 | DCN |  |  | 5233 | -1.798 | -0.4092 | Yes |
| 108 | LRP1 |  |  | 5234 | -1.801 | -0.3968 | Yes |
| 109 | SNAI2 |  |  | 5241 | -1.903 | -0.3848 | Yes |
| 110 | CDH11 |  |  | 5245 | -1.943 | -0.3720 | Yes |
| 111 | TPM2 |  |  | 5247 | -1.967 | -0.3586 | Yes |
| 112 | PCOLCE |  |  | 5248 | -1.999 | -0.3448 | Yes |
| 113 | MGP |  |  | 5252 | -2.037 | -0.3314 | Yes |
| 114 | PDGFRB |  |  | 5253 | -2.045 | -0.3172 | Yes |
| 115 | DAB2 |  |  | 5261 | -2.217 | -0.3033 | Yes |
| 116 | PTX3 |  |  | 5262 | -2.217 | -0.2880 | Yes |
| 117 | FBN1 |  |  | 5263 | -2.234 | -0.2726 | Yes |
| 118 | COL6A2 |  |  | 5265 | -2.276 | -0.2571 | Yes |
| 119 | COL4A1 |  |  | 5266 | -2.279 | -0.2414 | Yes |
| 120 | CTHRC1 |  |  | 5270 | -2.394 | -0.2254 | Yes |
| 121 | SPP1 |  |  | 5272 | -2.446 | -0.2087 | Yes |
| 122 | COL5A1 |  |  | 5274 | -2.455 | -0.1920 | Yes |
| 123 | LUM |  |  | 5277 | -2.507 | -0.1751 | Yes |
| 124 | MYL9 |  |  | 5280 | -2.643 | -0.1572 | Yes |
| 125 | TAGLN |  |  | 5281 | -2.669 | -0.1388 | Yes |
| 126 | INHBA |  |  | 5282 | -2.697 | -0.1202 | Yes |
| 127 | FN1 |  |  | 5287 | -2.866 | -0.1012 | Yes |
| 128 | EDIL3 |  |  | 5290 | -3.285 | -0.0789 | Yes |
| 129 | COL3A1 |  |  | 5292 | -3.630 | -0.0541 | Yes |
| 130 | COL6A3 |  |  | 5293 | -3.722 | -0.0284 | Yes |
| 131 | COL1A1 |  |  | 5294 | -4.144 | 0.0002 | Yes |
Table: GSEA details [plain text format]

  

Fig 2: HALLMARK\_EPITHELIAL\_MESENCHYMAL\_TRANSITION: Random ES distribution      
 Gene set null distribution of ES for **HALLMARK\_EPITHELIAL\_MESENCHYMAL\_TRANSITION**
